# The impact of hyperbaric oxygen therapy on serological values of vascular endothelial growth factor (VEGF) and basic fibroblast growth factor (bFGF)

**DOI:** 10.1186/1746-160X-6-29

**Published:** 2010-12-22

**Authors:** Susanne Jung, Kai Wermker, Harald Poetschik, Thomas Ziebura, Johannes Kleinheinz

**Affiliations:** 1 Department of Cranio-Maxillofacial Surgery, Research Unit Vascular Biology of Oral Structures (VABOS), University Hospital Muenster, Waldeyerstrasse 30, D-48149 Muenster, Germany; 2 Private practice, Lueneburg, Germany; 3 Department of Orthodontics, University Hospital Muenster, Germany

## Abstract

**Background:**

Hyperbaric oxygen (HBO) therapy is an effective adjunct treatment for ischemic disorders such as chronic infection or chronic wounds. It combines hyperoxic effects with the stimulating potential of post-therapeutic reactive hypoxia. As its crucial effects, stimulation of fibroblast growth, induction of collagen synthesis and the initiation of angiogenesis are discussed. Angiogenesis is a multistage process resulting in the growth of blood vessels. It includes degradation of extracellular matrix, proliferation and migration of different cell populations and finally formation of new vessel structures. This complex chain of procedures is orchestrated by different cytokines and growth factors. Crucial mediators of angiogenesis are basic fibroblast growth factor (bFGF) and vascular endothelial growth factor (VEGF); their *in-vivo *function is still not fully understood.

**Methods:**

Forty-three patients suffering from sudden sensorineural hearing loss or tinnitus were treated with HBO. The therapy included 10 sessions of 90 minutes each, one session a day. Serological levels of bFGF and VEGF were assessed by enzyme-linked immunosorbent assays performed according to the manufacturer's instructions on day 1, 2, 5 and 10 of HBO therapy and were compared to mean values of the control group, related to the patient's age and sex, and their development observed over the ten days of HBO.

**Results:**

There was no sex- or age dependency of bFGF observed in the present study, whereas under HBO our results showed a significant mitigation of the bFGF concentration. In the present data, there was no connection between the VEGF concentration and the patients' ages. Women showed significantly higher levels of VEGF. There was no significant change of VEGF concentration or the VEGF/bFGF ratio during HBO. All scored results varied within the range of standard values as described in the current literature.

**Conclusions:**

A significant effect of HBO on serum concentrations of bFGF and VEGF was not verified in the present study. Additional application of exogenous growth factors in conjunction with HBO was not obviously linked by a coherent cause-and-effect chain as far as wound healing is concerned.

## Background

Therapeutic administration of HBO was first mentioned in 1873 when miners were treated for decompression sickness [1]. Today, hyperbaric oxygen therapy is an effective treatment modality in the management of a variety of disorders, such as severe anaemia, gas gangrene, arterial gas embolism, carbon monoxide poisoning, radiation injuries, necrotizing infections, refractory wounds and chronic osteomyelitis but also acute ischemic disorders like acute sensorineural hearing loss or ischemic stroke [2].

Hyperbaric oxygen therapy involves the intermittent inhalation of 100% oxygen in chambers pressurized between 1.5 and 3.0 atmosphere absolute (ATA). An ATA is defined as the atmospheric pressure at sea level and is equivalent to 101.3 kilopascals or about 14.7 pounds per square inch. Its immediate effect is to increase dissolved oxygen content to above physiologic levels according to Krogh Erlang mechanism [3]. The benefit of HBO is based on the premise that raising tissue oxygen levels will enhance wound healing ability. The ignition for healing processes and angiogenesis is the lack of oxygen, but a sufficient oxygen supply is the basis for all proliferation or healing activities.

The positive effects of hyperoxia basically are

- vasoconstriction of arterioles, reduction of oedema

- bactericidal impact on anaerobial microbes and toxin-deactivation

- mobilisation and activation of leucocytes and macrophages

- activation of fibroblasts' collagen release

- activation of osteoclasts and stimulation of osteogensis

- cover of raised oxygen requirement of the damaged tissue in spite of impaired blood supply via stimulation of angiogenesis [4,5].

A final destination of all sorts of repair process is the re-establishment of a sufficient perfusion and oxygen supply. The present study focuses on the relevant mediators of angiogenesis which are vascular endothelial growth factor (VEGF) and basic growth factor (bFGF). In angiogenesis, a number of growth factors (for example Angiopoietin-1, Platelet derived growth factor, Hypoxia inducing factor-1α, EphrinB2, Nitric Oxygen Synthase) have been found to play significant roles; VEGF and bFGF are the most extensively investigated angiogenic factors to date [6,7].

As a single polypeptide bFGF is produced by a variety of cell populations, mainly by activated macrophages and thrombocytes. It belongs to one of the 22 members of the FGF family and transmits its signals via tyrosine kinase receptors [8,9]. Due to its ability to stimulate the activity of fibroblasts, endothelial cells, smooth muscle cells and neurons, bFGF is involved in many physiological and pathophysiological processes like growth, wound and bone healing, cell differentiation and proliferation, but also tumour progression and metastasis [10,11]. The key ability of bFGF is to induce angiogenesis via stimulation of VEGF expression, the effectiveness of a combined application is therefore easily explained. It is mitogenic on fibroblasts and endothelial cells [12].

VEGF is homodimer glycoprotein, its family includes 6 related proteins; VEGF 165 is most common and biologically active [13]. It is released by many cell populations as fibroblasts, monocytes, macrophages or lymphocytes [14]. The corresponding receptors belong to the tyrosine kinase family. VEGF induces angiogenesis on different levels: it acts as mitogen especially on endothelial cells, raises the vessel permeability and dilatation by releasing NO and has chemotactic impact on other growth promoting cell populations [15].

Under hypoxia an increase of VEGF mRNA could be shown, and in addition to that the RNA's half-life was extended. This effect is translated by the hypoxia sensitive transcription factor HIF-1. The instantaneous angiogenic effect of VEGF is the increase in vessel permeability and mitogenic stimulation of endothelial cells. According to its potential, VEGF is also involved in pathophysiological processes like tumour growth; mainly in hypoxic tumour regions raised VEGF levels could be scored [16,17].

The idea of the present study was to characterize and to quantify the effect of HBO on the expression of the growth factors bFGF and VEGF and, hence, to explain the mechanism of its therapeutic use.

## Methods

Between May and October 1999, specimens were obtained from 86 persons: 43 patients and 43 control subjects (Table 1). In the HBO group, 43 patients treated for acute hearing loss or tinnitus were enrolled. During 10 days, they received HBO for 90 minutes each day, the applied pressure was 1.55 bar.

**Table 1:** Patient data

	n	sex	age	average	SD	diagnosis
HBO group	43	22 ♂, 21 ♀	16-62 years	43.3 years	11.4 years	28 acute hearing loss 15 tinnitus
Control group	43	19 ♂, 24 ♀	18-65 years	29.9 years	14.1 years	-

Distribution of sex and age in the study and the control group (SD = standard deviation)

Blood samples of 7.5 ml of peripheral blood were taken on day 1, 2, 5 and 10 of HBO treatment, after examination and informed consent of the control persons using standard serum test tubes, respectively; samples were stored at 2-5°C, centrifuged (1000/min) after a 60-minute period of coagulation and stored at -80°C until analysis.

VEGF concentrations were assessed by performing a solid-phase VEGF Immunoassay (VEGF Quantikine, DVE00, R&D Systems GmbH, Wiesbaden-Nordenstadt, Germany R&D Systems). The ELISA was performed according to the manufacturer's protocol; its sensitivity was described as < 25 pg/ml. The concentration of VEGF was expressed as pg/ml. VEGF was quantified by using a standard curve made by human VEGF ranging from 62 pg/ml to 707 pg/ml. The chromogenic reaction was read at 450 nm.

The bFGF concentrations were assessed by performing a solid-phase bFGF Immunoassay (FGF basic Quantikine, HSFB50, R&D Systems GmbH, Wiesbaden-Nordenstadt, Germany R&D Systems). The ELISA was performed according to the manufacturer's protocol; its sensitivity was described as < 5 pg/ml. The concentration of bFGF was expressed as pg/ml. The bFGF was quantified by using a standard curve made by human bFGF ranging from 0 pg/ml to 4.40 pg/ml. The chromogenic reaction was read at 490 nm.

### Evaluated parameters

The evaluation focused on the most important angiogenic factors VEGF and bFGF and their serological values (Table 2) [18,19].

**Table 2:** Characteristics of bFGF and VEGF

	gene	weight	receptor	synthesis	standard value pg/ml
bFGF	5p12-p13	18 kDa	tyrosine kinase	macrophages	2.45
VEGF	6p12-p21	39-45 kDa	tyrosine kinase	fibroblasts	224

Characteristics of bFGF and VEGF

### Statistical analysis

The results are expressed as mean values +/- standard deviations (SD) for each group. Normal curve of distribution of results, means and SD were controlled with the David, Pearson and Stephens test. Data were statistically analyzed by using the analysis of variance (ANOVA) and Scheffe test. Differences between groups were assessed using the Whitney Mann U-test. An error probability of p < 0.05 was adopted as the statistically significant level.

## Results

### bFGF

There was no significant difference in bFGF concentration between HBO and control group before HBO. There was no sex- or age dependency according to our results. In the present study, under HBO a significant mitigation of the bFGF concentration within standard-values could be observed (Table 3). Differences between bFGF concentrations at day 1 and day 10 within the HBO group reached statistical significance (p = 0.041).

**Table 3:** Serological values of bFGF

	bFGF in pg/ml	
	mean	SD
Control group	2.65	3.01
HBO group - day 1	3.25	2.38
day 2	2.80	1.94
day 5	2.55	1.72
day 10	2.12	1.23

Serological values of bFGF in the study and the control group at definite times

### VEGF

There was a significant difference in VEGF concentration between patients and control group before HBO (p = 0.015). In our results there was no connection between the VEGF concentration and the patients' ages. Women showed significantly higher levels of VEGF (279.65 +/- 243.70 pg/ml) compared to male subjects (193.58 +/- 162.39 pg/ml) (p = 0.026). There was no significant change of VEGF concentration under HBO (Table 4).

**Table 4:** Serological values of VEGF

	VEGF in pg/ml	
	mean	SD
Control group	190.63	175.27
HBO group - day 1	282.60	252.87
day 2	290.16	248.35
day 5	269.89	224.18
day 10	279.58	263.24

Serological values of VEGF in the study and the control group at definite times

### VEGF/bFGF ratio

**Figure 1:**
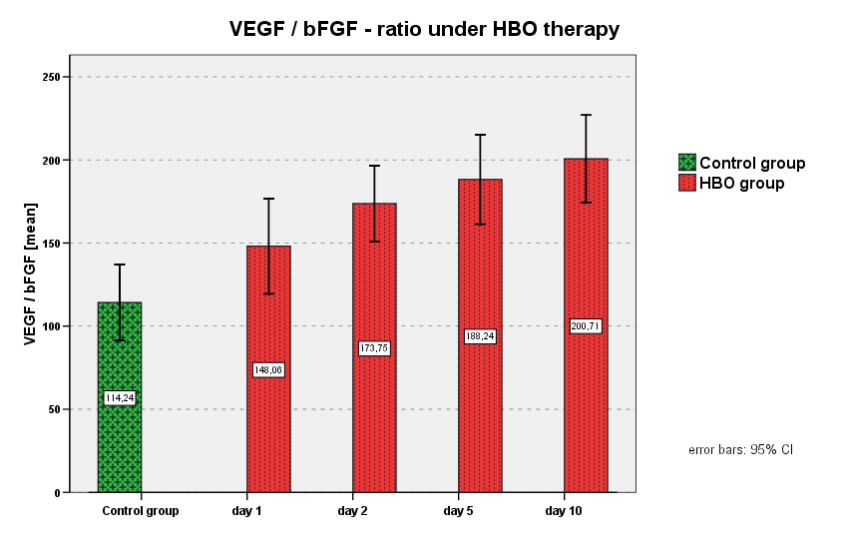
**VEGF/bFGF ratio under HBO**.

Although a slight increase of the ratio could be documented, there was no significant variance of the VEGF/bFGF ratio in the present study (Figure 1).

## Discussion

Under HBO therapy a significant reduction of bFGF was detected. The concentration of VEGF did not change but was measured significantly higher in female test persons. All collected results were within the range of standard values according to the current literature.

The influence of the ischemic inner-ear disorder for which the HBO group was treated could not be fully excluded as an interfering factor. Yet it seemed highly implausible because so far only grave diseases have been reported to affect bFGF and VEGF concentration [20]. There are several explanations for the reduced bFGF level under HBO. One hypothesis states that there are simply more bFGF molecules binding on their receptor and transmitting their signal. These molecules are no longer detectable for the performed assays.

There might be a higher rate of binding on circulation proteins in the course of repair processes: these ligand-receptor complexes are possibly the activating agents inducing fibroblast proliferation, collagen release, osteo- and angioneogenesis.

Eventually, a therapy period of ten days was observed. Possibly, there was a delayed cellular adaptation to the HBO treatment, where protective cell populations have to be activated for repair mechanisms to oxygen free-radical damage. There are hints in literature that effective cellular responses to HBO cannot be expected before day seven of treatment [21].

Another consideration is that acute sensoneurinal hearing loss and tinnitus do not require the induction of wound healing algorithm to a comparable extend as tissue lesions or chronic infections do. So the restraint reaction of bFGF and VEGF in our data might be explained.

The observed higher levels of VEGF in females find their explication in their cycle-dependent hormone regulation.

As far as VEGF concentration during HBO is concerned our data suggests that the reactive hypoxia was not enough of a stimulus for increased VEGF release. Yet *in-vivo *and *in-vitro *studies have underlined the fact that hypoxia is a potent stimulus for VEGF release [22]. In malignantly transformed tissues the highest rates of VEGF were found in hypoxic, necrotic areas [23].

Application of recombinant human VEGF showed promising results in animal studies by promoting collateral formation in occluded vessels or proliferation of endothelial cells in severed arteries [24].

A not yet mentioned field of interest dealing with VEGF lies in tumour diagnosis and follow-up care. VEGF might be able to act as an indicator of tumour neo angiogenesis for elevated VEGF levels are observed and even correlate with the tumour mass in patients with malignant melanoma [25].

Some tumour entities as sarcomas are not accompanied by elevated tumour markers like PSA in prostate cancer [26,27]. As soon as VEGF's role in tumour growth, in metastasis and, in particular, tumour neo-angiogenesis will be further elucidated, VEGF serum level and its development during the etiopathology might become valuable prognostic tools [28,29].

## Conclusions

Although in the present data a significant effect of HBO on serum concentrations of bFGF and VEGF was not verified, many studies revealed that intermittent HBO exposure plays a direct role in vascular growth; the biochemical processes, however, have not been fully understood. Additional application of exogenous growth factors in conjunction with HBO was not obviously linked by a coherent cause-and-effect chain as far as wound healing is concerned.

## Competing interests

The authors declare that they have no competing interests.

## Authors' contributions

SJ carried out the immunoassays and drafted the manuscript, KW and SJ participated in the design of the study and performed the statistical analysis. HP and JK conceived of the study, and participated in its design and coordination and helped to draft the manuscript. HP and TZ were involved in revising the article and all authors read and approved the final manuscript.
